# A Longitudinal Study of Thrombosis and Bleeding Outcomes With Thromboprophylaxis in Pregnant Women at Intermediate and High Risk of VTE

**DOI:** 10.1177/10760296231160748

**Published:** 2023-03-27

**Authors:** E. Schapkaitz, E. Libhaber, A. Gerber, H. Rhemtula, J. Zamparini, B.F Jacobson, H.R Büller

**Affiliations:** 1 Department of Molecular Medicine and Hematology, University of Witwatersrand Medical School, Johannesburg, South Africa; 2 Department of Research Methodology and Statistics, University of Witwatersrand Medical School, Johannesburg, South Africa; 3 Department of Obstetrics, University of Witwatersrand Medical School, Johannesburg, South Africa; 4 Department of Internal Medicine, University of Witwatersrand Medical School, Johannesburg, South Africa; 5 Department of Vascular Medicine, 1234 University of Amsterdam, Amsterdam, the Netherlands

**Keywords:** pregnancy, low molecular weight heparin, venous thromboembolism, thromboprophylaxis, bleeding

## Abstract

**Background:**

The efficacy and safety of thromboprophylaxis in pregnancy at intermediate to high risk of venous thrombo-embolism (VTE) is an area of ongoing research.

**Aim:**

This study aimed to assess thrombosis and bleeding outcomes associated with thromboprophylaxis in women at risk of VTE.

**Methods:**

A cohort of 129 pregnancies, who received thromboprophylaxis for the prevention of VTE, were identified from a specialist obstetric clinic in Johannesburg, South Africa. Intermediate-risk pregnancies, with medical comorbidities or multiple low risks, were managed with fixed low-dose enoxaparin antepartum and for a median (interquartile range) of 4 (4) weeks postpartum. High-risk pregnancies, with a history of previous VTE, were managed with anti-Xa adjusted enoxaparin antepartum and for a median of 6 (0) weeks postpartum. Pregnancy-related VTE was objectively confirmed. Major bleeding, clinically relevant nonmajor bleeding (CRNMB) and minor bleeding were defined according to the International Society on Thrombosis and Hemostasis Scientific Subcommittee.

**Results:**

Venous thrombo-embolism occurred antepartum in 1.4% (95% CI: 0.04-7.7) of intermediate and 3.4% (95% CI: 0.4-11.7) of high-risk pregnancies. Bleeding events occurred in 7.1% (95% CI: 2.4-15.9) of intermediate and 8.5% (95% CI: 2.8-18.7) of high-risk pregnancies. Of these bleeding events, 3.1% (95% CI: 1.0-8.0) were classified as major bleeding. On univariate analysis, no independent predictors of bleeding were identified.

**Conclusion:**

The rates of thrombosis and bleeding in this predominantly African population were consistent with similar studies and can be used to inform pregnant women of the benefits of anticoagulation and the risks of potential bleeding.

## Introduction

Venous thrombo-embolism (VTE), which comprises deep vein thrombosis (DVT) and its complication (PE), is an important cause of pregnancy-related morbidity and mortality.^1–4^ Worldwide, the prevalence differs considerably, with a higher reported frequency in populations of African ethnicity.^2,5–8^ The physiological changes of pregnancy are associated with a background risk for VTE. In addition, multiple risk factors which further increase the risk of VTE have been identified by a number of high-quality cohort and case-control studies.^6,9–13^ In particular, a personal history of thrombosis and inherited thrombophilia have both been shown to be important risk factors for pregnancy-related VTE.^6,14–16^ The further increased risk among pregnant women of African ethnicity has been attributed to medical comorbidities including; peripartum cardiomyopathy, sickle cell disease, and human immunodeficiency virus (HIV) infection.^4,17,18^ HIV is of particular concern considering the increased prevalence among women in the reproductive age group as it confers at least a 2-fold increased risk of VTE.^19^ These and other clinical risk factors, such as maternal obesity and multiparity, have been associated with a low to intermediate risk of VTE.^6,11,13^

Antepartum and postpartum thromboprophylaxis with low molecular weight heparin (LMWH) is recommended for the prevention of pregnancy-related VTE in women at a sufficiently high risk of thrombosis.^20,21^ Nonetheless, the efficacy and safety of thromboprophylaxis are limited by a paucity of randomized controlled studies in women at an increased risk of VTE.^22–24^ A recent systematic Cochrane review found insufficient evidence to make recommendations for thromboprophylaxis.^25^ In this meta-analysis of 4 randomized controlled studies in 476 women, the use of antepartum heparin versus no treatment or placebo was not associated with a decreased VTE risk. The rates of bleeding in these studies were variably reported and thereby limited precise safety estimates of thromboprophylaxis.

Several research groups have carried out observational studies of thrombosis and bleeding outcomes associated with thromboprophylaxis in pregnant women. An earlier cohort study from the Netherlands evaluated 91 women at high and intermediate risk of VTE who received low-dose nadroparin.^26^ Low-dose thromboprophylaxis was associated with recurrent VTE in women at high risk with a postpartum incidence of 7.0% (95% CI: 2.9-16.7) and an antepartum incidence of 1.8% (95% CI: 0.4-9.2). The rate of severe postpartum hemorrhage (PPH) (defined as blood loss of >1 000 mL) was 9.1% (95% CI: 4.7-16.9) and 6 women received transfusions. Recently, Cox et al published a 15-year cohort study of 123 women at intermediate to high risk for VTE who received low-dose enoxaparin thromboprophylaxis.^27^ The rate of VTE was 1.2% (95% CI: 0.32-4.14) and these events occurred in the antepartum period. The rate of severe PPH was 9.3%, with these events occurring following cesarean delivery. Most recently, Rajaratnam et al have reported on their 10-year experience in 409 women at King's College Hospital.^28^ In these women who received weight-adjusted enoxaparin thromboprophylaxis, the rate of VTE was 1.4% and the rate of severe PPH was 14.1%. These studies, however, applied inconsistent definitions of bleeding in pregnancy and the postpartum period. Furthermore, there is limited evidence for thromboprophylaxis among African women, in particular those with intermediate and multiple low-risk factors.^29,30^ Further research is required to determine the balance of pregnancy-related thrombosis and major bleeding risks in this pregnant population.

In view of these considerations, we conducted a descriptive longitudinal study at a single academic center in a cohort of pregnant women at intermediate and high risk of VTE to assess the thrombosis and bleeding outcomes associated with antepartum and postpartum thromboprophylaxis.

## Methods

### Study Design and Population

A retrospective cohort study was conducted at the combined cardiac obstetric clinic at Charlotte Maxeke Johannesburg Academic Hospital, a quaternary-level maternity service in South Africa. This is a specialist referral clinic for women with cardiovascular disease including; congenital heart disease, pulmonary hypertension, coronary artery disease, cardiomyopathy, heart failure, arrhythmias, and VTE. Approximately 90 to 100 women attend this clinic per year. All women requiring antepartum thromboprophylaxis are managed by a multi-disciplinary team at the combined cardiac obstetric clinic. The clinical records of consecutive pregnant women, who received enoxaparin thromboprophylaxis for the prevention of VTE between March 1, 2017 and September 1, 2022, were reviewed. During the study period, 226 clinical records were identified. The study included 129 pregnancies which were classified as a high or intermediate risk for VTE according to local recommendations (Table 1). The intermediate-risk group included pregnancies with a high-risk inherited thrombophilia, antiphospholipid syndrome, medical comorbidities, or multiple low-risk factors (Table 2). Medical comorbidities included cardiomyopathy, heart failure, active systemic lupus erythematosus (SLE), pulmonary arterial hypertension, sickle cell disease, inflammatory polyarthropathy, and nephrotic syndrome. Peripartum and dilated cardiomyopathy with left ventricular (LV) ejection fraction < 40%, were considered for thromboprophylaxis in the presence of one or more of the following risk factors: atrial fibrillation, LV thrombus, and/or systemic embolism.^31^ Multiple low-risk factors included > 4 antepartum or ≥ 2 postpartum risk factors, namely age > 35 years, body mass index ≥ 30 kg/m^2^, parity ≥ 3, gross varicose veins, current systemic infection, paraplegia, pre-eclampsia with intra-uterine growth restriction, multiple pregnancies and human immunodeficiency virus-associated stroke.^32^ The high-risk group consisted of pregnancies with a history of clinical and radiological evidence for previous VTE. VTE was classified as unprovoked (in the absence of risk factors), provoked (within 3 months of a risk factor) or pregnancy or estrogen-related. Women who received thromboprophylaxis for the prevention of thrombo-embolism for valvular heart disease, congenital heart disease, arrhythmia, recurrent pregnancy loss, previous superficial venous thrombosis, and transient risk factors were excluded (n = 97). The study was approved by the Wits Human Research Ethics Committee, Medical (M-220207).

**Table 1.** Recommended Risk Stratification for Venous Thrombo-Embolism at Charlotte Maxeke Johannesburg Academic Hospital.^32^

Antepartum Risk Factors	Postpartum Risk Factors	Recommendation
**High risk**		
Previous unprovoked or pregnancy or estrogen-related VTE	Anyone requiring antepartum prophylaxis Previous VTE High-risk inherited thrombophilia* Low-risk inherited thrombophilia and family history** Anti-phospholipid syndrome	Antepartum thromboprophylaxis Postpartum thromboprophylaxis for at least 6 weeks
**Intermediate risk**		
Single VTE related to a transient risk factor (not related to pregnancy or estrogen use). High-risk inherited thrombophilia* Nonobstetric surgery during pregnancy Medical comorbidities*** Antiphospholipid syndrome	Cesarean delivery in labor Readmission or prolonged admission (≥ 3 days) postpartum Surgery in the puerperium Medical comorbidities*** BMI ≥ 40 kg/m^2^	Consider antepartum thromboprophylaxis Postpartum thromboprophylaxis for at least 2 weeks. If risk factors persist, consider extending thromboprophylaxis
**Low Risk**		
Low-risk inherited thrombophilia** Age > 35 years BMI ≥ 30 kg/m^2^ Parity ≥ 3 Smoker Family history of unprovoked or estrogen-related VTE in first-degree relative Gross varicose veins Current systemic infection Immobility Current pre-eclampsia with intra-uterine growth restriction Multiple pregnancies In vitro fertilization Dehydration/hyperemesis	Age > 35 years BMI ≥ 30 kg/m^2^ Parity ≥ 3 Smoker Elective cesarean delivery Family history of VTE Low-risk inherited thrombophilia Gross varicose veins Current systemic infection or peri-operative infection Immobility Multiple pregnancies Preterm delivery (< 37 weeks) Stillbirth Mid-cavity or rotational operative delivery Prolonged labor (> 24 h) Postpartum hemorrhage (> 1 000 ml and surgery or blood transfusion)	Consider antepartum thromboprophylaxis if multiple (>4) risk factors present Postpartum thromboprophylaxis at least until discharge from hospital if multiple (≥2) risk factors present

Abbreviations: VTE, venous thrombo-embolism; BMI, body mass index.

*High-risk inherited thrombophilia: antithrombin deficiency, compound heterozygous or homozygous for low-risk thrombophilia.

**Low-risk inherited thrombophilia: protein S deficiency, protein C deficiency, heterozygous for Factor V Leiden or prothrombin gene G20210A mutations.

***Medical comorbidities: cancer, heart failure, active systemic lupus erythematosus, inflammatory polyarthropathy or inflammatory bowel disease; nephrotic syndrome; type 1 diabetes mellitus with nephropathy; sickle cell disease; current intravenous drug user.

**Table 2.** Indications for Thromboprophylaxis in Intermediate- and High-Risk Pregnancies (n = 129).

Indications for Thromboprophylaxis	n (%)
**Intermediate-risk pregnancies for VTE**	**70 (54.2)**
**Medical comorbidities**	**50 (71.4)**
Cardiomyopathies and heart failure*	24 (48.0)
Active systemic lupus erythematosus	14 (28.0)
Pulmonary arterial hypertension**	7 (14.0)
Sickle cell disease	3 (6.0)
Inflammatory polyarthropathy	2 (4.0)
Nephrotic syndrome	1 (2.0)
Multiple low-risk factors***	**20 (28.6)**
**High-risk pregnancies for VTE**	**(59, 45.7)**
**Personal history of VTE**	**59 (45.7)**
Unprovoked VTE	33 (55.9)
Pregnancy or estrogen-related VTE	23 (39.0)
Provoked VTE****	3 (5.1)
Recurrent VTE	10 (16.9)
Antiphospholipid syndrome with thrombosis	2 (3.4)
Protein S deficiency with thrombosis*****	9 (17.3)
Protein C deficiency with thrombosis*****	2 (3.8)

Abbreviations: VTE, venous thrombo-embolism.

^*^ Peripartum and dilated cardiomyopathy and left ventricular (LV) ejection fraction < 40%, or atrial fibrillation, or LV thrombus, or systemic embolism.

**World Health Organization (WHO) Group I = 3, WHO Group II = 1, WHO Group III = 2, WHO Group IV = 1.

***Including: Age > 35 years, body mass index ≥ 30 kg/m^2^, parity ≥ 3, gross varicose veins, current systemic infection, paraplegia, pre-eclampsia with intra-uterine growth restriction, multiple pregnancies, human immunodeficiency virus-associated stroke.

****Provoked and recurrent (n = 1), provoked and multiple low risk factors (n = 2).

*****Testing for proteins S and C in 52 high-risk pregnancies.

### Study Protocol

#### Data collection

Maternal demographics, obstetric and delivery characteristics were recorded. In the women living with HIV, data on HIV diagnosis, antiretroviral therapy (ART) and adherence were also recorded.

#### Management

VTE risk assessment was performed at the first antepartum visit and repeated postpartum, following delivery and prior to discharge. Seventy pregnancies were classified as intermediate risk and 59 pregnancies were classified as high risk, antepartum as well as postpartum. Women at intermediate risk for VTE, were managed with fixed low dose daily subcutaneous enoxaparin (according to weight namely, 20 mg for <50 kg; 40 mg for 50-90 kg; 60 mg for 91-130 kg; and 80 mg for 131-170 kg) antepartum and continued for at least 2 weeks postpartum (Table 3).^32^ Thromboprophylaxis was extended in the presence of persistent risk factors. In those considered to be at high risk for VTE, enoxaparin was prescribed antepartum and continued until 6 weeks postpartum. Doses were adjusted with regular anti-Xa monitoring to achieve a prophylactic anti-Xa level of 0.2-0.6 units/mL. There were 9 (7.0%) pregnancies which were switched from extended duration anticoagulation at pregnancy diagnosis to enoxaparin 12-hly, adjusted to achieve a therapeutic anti-Xa level of 0.6-1.0 units/mL.

**Table 3.** Antepartum, Peripartum, and Postpartum Management of Intermediate- and High-Risk Pregnancies.

	Intermediate Risk for VTE	High Risk for VTE
**Number of pregnancies**	70	59
**Antepartum**		
Gestational age enoxaparin commenced (weeks), median (IQR)*	24 (13)	12 (14)
Prophylactic daily enoxaparin dose, median (IQR)**	40 (20)	80 (40)
Therapeutic twice daily enoxaparin dose, median (IQR)**	-	80 (10)
Concomitant aspirin, 75-150 mg (n, %)***	22 (31.4)	13 (22.0)
Peripartum****		
Last enoxaparin injection to delivery interval (hours), median (IQR)	28 (22)	24 (24)
First postpartum enoxaparin injection post-delivery interval (hours), median (IQR)	15 (12)	13 (11)
Postpartum****		
Duration of postpartum enoxaparin (weeks), median (IQR)	4 (4)	6 (0)

Abbreviation: IQR, inter-quartile range; VTE, venous thrombo-embolism.

*In women receiving extended-duration anticoagulation; a switch was made to enoxaparin at a median of 8 (6) weeks’ gestation.

**prophylactic enoxaparin n = 120; therapeutic enoxaparin n = 9.

In the women receiving therapeutic enoxaparin, enoxaparin was discontinued at a median of 18 (16) prior to delivery and reintroduced at 13 (28) h after delivery. In the women receiving prophylactic enoxaparin, enoxaparin was discontinued at a median of 24 (18) h prior to delivery and reintroduced at 16 (12) h after delivery.

***Prior to 16 weeks gestation until delivery.

****Of live births.

#### Outcomes

Pregnancy-related VTE was defined as VTE during pregnancy or 3 months postpartum. Symptomatic VTE was conﬁrmed by compression ultrasound or computed tomography pulmonary angiogram. Postpartum hemorrhage referred to bleeding by visual estimation during the first 24 h (primary) or from 24 h to 6 weeks after delivery (secondary).^33^ Major bleeding, clinically relevant nonmajor bleeding (CRNMB), and minor bleeding events were confirmed by an independent adjudication committee (ES, HR, and JZ).^34,35^

#### Laboratory methods

Venous blood for anti-Xa measurement was collected in 3.2% sodium citrate (Becton-Dickinson, Oxford, the United Kingdom) at a mean ± standard deviation (SD) of 3.3 ± 0.4 h after the last enoxaparin injection. Anti-Xa analysis was performed on the STA-R Max^®^ automated coagulation analyzer (Diagnostica Stago, Asnières sur Seine, France). Serial peak anti-Xa levels were available in 52 (88.1%) high-risk pregnancies. With pregnancy progression, 10 (19.2%) required a dose adjustment in the second trimester and 13 (25.0%) in the third trimester to achieve the target range.

### Statistical Methods

A sample of 126 pregnancies was estimated, assuming a 2.0% incidence of bleeding or VTE during pregnancy and the postpartum period and considering a maximum incidence of 15%, at a confidence interval (CI) of 95% (Epi Info v7.2.0.1, Atlanta, Georgia, USA).^36^ The statistical unit of analysis was pregnancy. Clinical and laboratory data was analyzed using Statistica 13.2 software (Palo Alto, California, the United States). Normally distributed continuous data are presented as mean ± SD and variables with non-Gaussian distribution as median (interquartile range [IQR]). Categorical data are presented as frequencies and percentages. Comparisons for continuous measurements were performed using a one-way ANOVA test with Sheffes's post hoc test or a Kruskal-Wallis test. Categorical variables were compared using chi-square test or Fisher's exact test. The median time to VTE, major bleeding, and CRNMB were estimated using the Kaplan–Meier method. Univariate Cox Proportional Hazard Regression analysis was used to identify predictors of bleeding events by calculating hazard ratios (HR with 95% CI). Statistical significance was set at a *P* value of <.05.

## Results

### Patient Characteristics

A total of 129 pregnancies in 127 women were eligible for inclusion. The characteristics of the 70 (54.3%) intermediate-risk and 59 (45.7%) high-risk pregnancies for VTE are described in Table 4. The majority were of African ethnicity (n = 123, 95.3%) in keeping with the demographics of the population served by our hospital. Thirty-five (27.1%) were living with HIV with a CD4 count of 501 (375)×10^6^/L. The majority (n = 26, 74.3%) were virologically suppressed on first-line ART for a median duration of 5 (6) years. Adverse obstetric characteristics: chronic hypertension, pre-eclampsia with fetal growth restriction (FGR) and prolonged hospital admission were significantly increased in the intermediate-risk group whereas miscarriage was significantly increased in the high-risk group.

**Table 4.** Characteristics According to Risk Group.

Characteristics	Intermediate Risk for VTE	High Risk for VTE	*P* Value
**Number of pregnancies**	70	59	
**Demographics**			
Age at study entry (years), mean ± SD	32.8 ± 5.7	32.6 ± 5.4	.800
BMI (kg/m^2^), mean ± SD	29.6 ± 8.3	31.1 ± 6.1	.242
Parity, median (IQR)	2.0 (2)	2.0 (2)	.969
HIV infected (n, %)	18 (25.7)	17 (28.8)	.693
**Baseline laboratory characteristics**			
White cell count (× 10^9^/L), mean ± SD (ref: 3.9-12.6)	8.1 ± 3.1	7.2 ± 1.9	.046
Hemoglobin (g/L), mean ± SD (ref: 116-164)	119.1 ± 16.2	121.8 ± 13.0	.312
Platelet count (× 10^9^/L), median (IQR) (ref: 186-454)	228.0 (112.0)	242.0 (87.0)	.618
eGFR (mL/min/1.73 m^2^ ), median (IQR) (ref: > 60)	60 (0)	60 (0)	1.000
**Obstetric characteristics**			
Chronic hypertension (n, %)	24 (34.3)	7 (11.9)	.003
Pre-eclampsia and FGR (n, %)*	8 (11.8)	1 (2.0)	.048
Systemic infection (n, %)	6 (8.6)	3 (5.1)	.439
Prolonged hospital admission (n, %)	17 (24.3)	6 (10.2)	.037
Multiple pregnancy (n, %)	2 (2.9)	2 (3.4)	.862
**Delivery characteristics**			
Live births (n, %)	66 (94.3)	50 (86.2)	.138
Intra-uterine fetal death (n, %)	4 (5.7)	0 (0.0)	.126
Miscarriage (n, %)	0 (0.0)	5 (8.6)	.017
Termination of pregnancy (n, %)	0 (0.0)	3 (5.2)	.090
Gestational age delivery (weeks), mean ± SD**	36.5 ± 2.7	37.7 ± 1.7	.009
Birthweight (g), mean ± SD**	2633.7 ± 768.0	2938.7 ± 549.3	.023
Scheduled delivery (n, %)	50 (75.8)	39 (78.0)	.515
Mode of delivery**			
Normal vaginal delivery (n, %)	20 (30.3)	21 (42.00)	.192
Elective cesarean delivery (n, %)	28 (42.4)	18 (36.0)	.484
Emergency cesarean delivery (n, %)	18 (27.3)	11 (22.0)	.516
Neuraxial anesthesia (%)***	30 (65.2)	27 (93.1)	.006
Estimated blood loss at delivery (mL), median (IQR)			
Normal vaginal delivery	200 (120)	250 (250)	.160
Cesarean delivery	500 (200)	500 (250)	.972

Abbreviations: SD, standard deviation, IQR, inter-quartile range; BMI, body mass index; HIV, human immunodeficiency virus; FGR, fetal growth restriction; eGFR, estimated glomerular filtration rate; VTE, venous thrombo-embolism.

*In 118 pregnancies

**Of live births

***Of cesarean deliveries

### Outcomes

**Figure 1.**
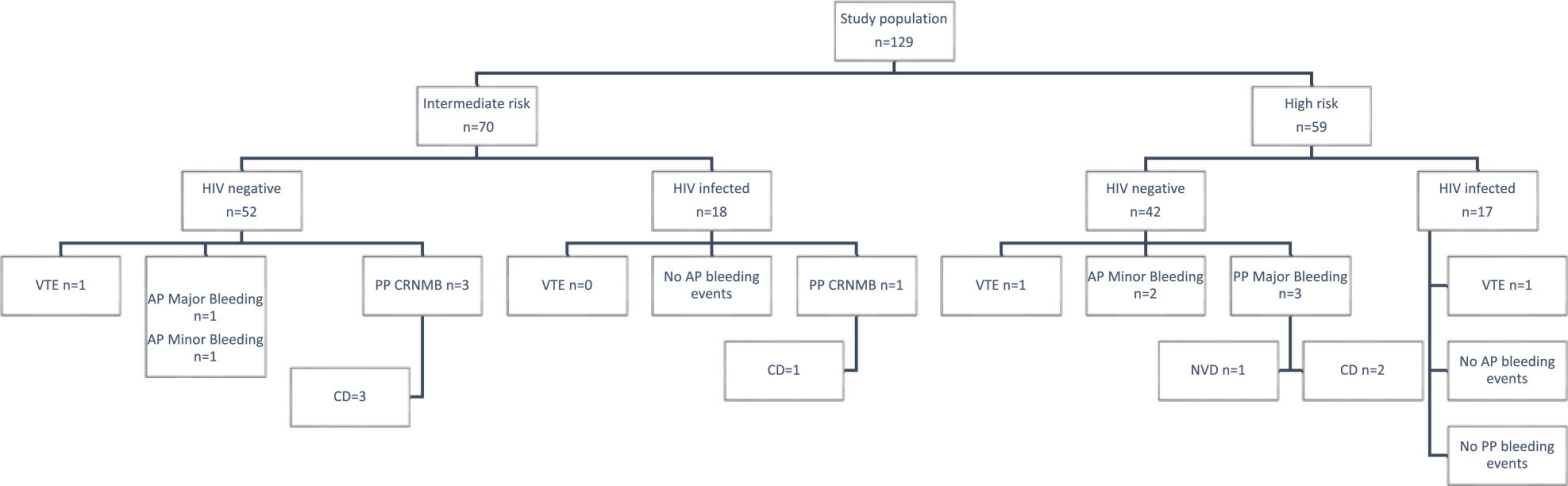
Schematic diagram of study outcomes.

The flow diagram in Figure 1 illustrates the thrombotic and major bleeding events in pregnancies at intermediate and high risk of VTE.

The incidence of antepartum VTE was 2.3% (95% CI: 0.5-6.9). In intermediate- and high-risk pregnancies, the incidence of VTE was 1.4% (95% CI: 0.04-7.7) and 3.4% (95% CI: 0.4-11.7), respectively. A fatal PE, occurred in a 31-year-old, HIV-negative woman, at 19 weeks’ gestation, with a personal history of postpartum PE and active SLE. She was receiving an inadequate dose of enoxaparin (anti-Xa = 0.1 IU/mL), the dose of which was increased 2 weeks prior to her event. Obstetric history was noteworthy for an intra-uterine fetal death at 30 weeks’ gestation associated with anticardiolipin and anti-beta2-glycoprotein IgG positivity on a single occasion. A left-leg DVT occurred in a 36-year-old woman living with HIV, at 23 weeks gestation, with a personal history of an unprovoked DVT. The patient was adherent to ART (Zidovudine, Lamivudine, and Aluvia) for 4 years with a CD4 count of 230 × 10^6^/L and HIV viral load of 622 copies/mL. She was receiving low-dose enoxaparin from 8 weeks gestation. Compression ultrasound showed an acute proximal DVT of the femoral-popliteal system. The patient was switched to anti-Xa adjusted therapeutic enoxaparin for the remainder of the pregnancy. In the postpartum period, the patient was transitioned to long-term warfarin. Lastly, a PE, occurred in a 26-year-old, HIV-negative woman, at 32 weeks gestation, with a personal history of active SLE and prolonged hospital admission for disease exacerbation and systemic infection. She was receiving fixed low-dose enoxaparin from 28 weeks gestation.

The incidence of bleeding was 7.1% (95% CI: 2.4-15.9) and 8.5% (95% CI: 2.8-18.7) in intermediate- and high-risk pregnancies, respectively. There were 4 antepartum and 6 postpartum bleeding events of which 4 were major bleeding events (3.1%; 95% CI: 1.0-8.0), 3 were CRNMB events (2.3%, 95% CI: 0.5-6.9) and 3 were minor bleeding events (2.3%, 95% CI: 0.5-6.9; Table 5). On univariate logistic regression analysis, no independent predictors of bleeding were found (Table 6). Pre-eclampsia with FGR could not be calculated as the assumptions of proportionality were not met.

**Table 5.** Description of Bleeding Events in Intermediate- and High-Risk Pregnancies.

	Description
**Intermediate risk**	
Major bleeding	Antepartum placenta praevia at 34 weeks
CRNMB	Multi-fibroid uterus and postpartum blood loss ≥1 000 mL and first line treatment with uterotonics
	Postpartum blood loss ≥1 000 mL and first line of treatment with uterotonics and tranexamic acid
	Uterine atony and postpartum blood loss ≥1 000 mL and first line of treatment with uterotonics
Minor bleeding	Antepartum vaginal bleeding prompting a face-to-face evaluation at 34 weeks
**High risk**	
Major bleeding	Postpartum blood loss ≥1 000 mL and transfusion and balloon tamponade
	Postpartum blood loss ≥1 000 mL leading to transfusion
	Postpartum blood loss <1 000 mL and transfusion
Minor bleeding	Antepartum vaginal bleeding prompting a face-to-face evaluation at 39 weeks
	Antepartum vaginal bleeding prompting a face-to-face evaluation at 29 weeks

Abbreviation: CRNMB, clinically relevant non major bleeding.

**Table 6.** Univariate Cox Proportional Hazard Regression Analysis of Predictors of Bleeding.

Variable	HR	95% CI	*P* Value
High-risk pregnancies	1.29	0.37-4.46	.687
HIV	0.30	0.04-2.34	.250
Concomitant aspirin	2.63	0.76-9.07	.127
Cesarean delivery	2.17	0.46-10.21	.327
Spontaneous delivery	0.39	0.05-3.08	.372
Peripartum enoxaparin interval <12 h prior to delivery	1.09	0.23-5.12	.916
Peripartum enoxaparin interval <12 h post delivery	1.12	0.32-3.88	.855

Abbreviations: HR, hazard ratio; CI, confidence interval; HIV, human immunodeficiency virus.

Assumptions of proportionality were not met for pre-eclampsia and fetal growth restriction.

Peripartum, spinal, or epidural anesthesia were administered in 30 (62.5%) intermediate-risk and 27 (87.1%) high-risk cesarean deliveries, respectively, with no bleeding or neurological complications. There was a single (0.8%) wound hematoma post-delivery. There were 7 (5.4%) cases of excessive bruising and no hypersensitivity skin reactions at the injection sites. No women switched to another type of LMWH.

**Figure 2.**
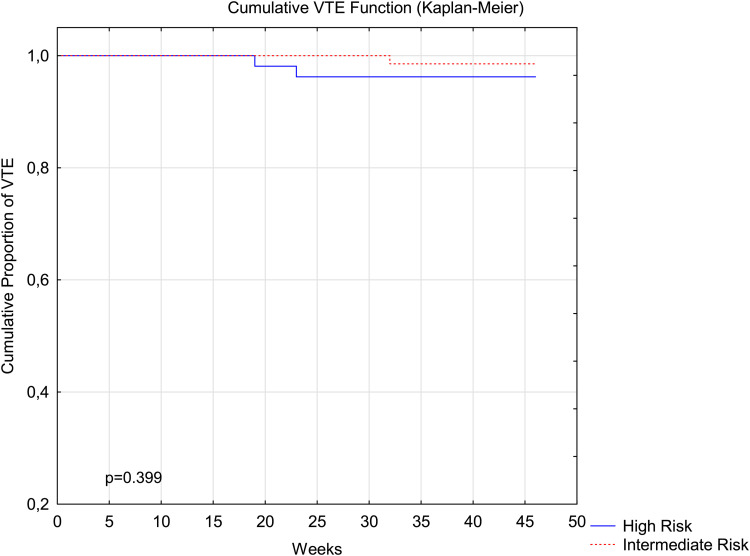
Thrombosis events in intermediate- and high-risk pregnancies. Kaplan–Meier curves are shown for the first occurrence of symptomatic venous thromboembolism in the overall study period.

**Figure 3.**
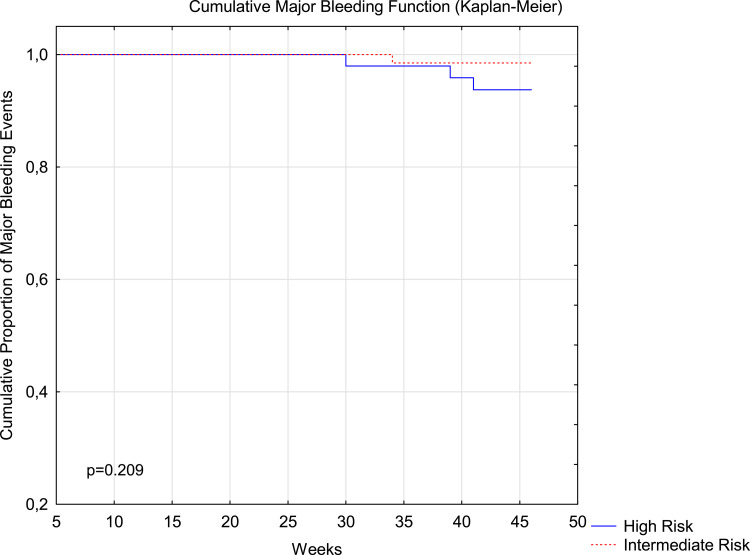
Major Bleeding events in intermediate- and high-risk pregnancies. Kaplan–Meier curves are shown for the first occurrence of adjudicated major bleeding events in the overall study period.

Figures 2 and 3 illustrate the timing of thrombotic and bleeding events, respectively, in intermediate- and high-risk pregnancies.

## Discussion

Low rates of VTE have been reported previously with the use of thromboprophylaxis for intermediate risk factors.^26–28,37–39^ In this cohort, more than half of the pregnancies were classified as intermediate risk, which were managed with fixed low-dose enoxaparin. The incidence of VTE was 1.4%. In contrast to previously published studies which included thrombophilia and single VTE related to a temporary risk factor, the indications for thromboprophylaxis in this cohort, of predominantly African ethnicity, were largely for medical comorbidities. Heart disease and active SLE in pregnancy are recognized risk factors for VTE which have been associated with at least a 6-fold increased risk of antepartum VTE. Similarly, sickle cell disease has been associated with at least a 4-fold increased risk of VTE.^6^ The incidence of sickle cell disease was, however, low in accordance with an earlier South African report.^40^ Moreover, in this study cohort, the risks were further heightened by the presence of high-risk obstetric and delivery characteristics, which is consistent with international cohorts.^27,41^ Studies to date have highlighted the wide variation in the associated risks of pregnancy-related thrombosis in pregnancies classified as intermediate risk.^6,11,14^ A randomized controlled trial is required to determine the safety and efficacy of postpartum and/or fixed low-dose antepartum thromboprophylaxis. This will assist in refining thromboprophylaxis indications and reducing the bleeding complications in intermediate-risk pregnancies.

Despite the use of anti-Xa-adjusted enoxaparin in pregnancies classified as high-risk, the incidence of thrombosis was 3.4%. In the majority of high-risk pregnancies, anti-Xa levels were monitored at regular intervals, in order to facilitate dose adjustments with advancing gestation. With pregnancy progression, 19.2% required a dose adjustment in the second trimester and 25.0% in the third trimester to achieve the target range. This supports prior observational studies.^37,38,42,43^ Owing to the low number of recurrent VTE events in high-risk pregnancies, the efficacy of anti-Xa monitoring could not be confirmed in this study. Regular anti-Xa monitoring too has several limitations. The timing of the assay is important for accurate interpretation and there is no established prophylactic range for enoxaparin in pregnancy.^44^ Furthermore, this specialized assay remains relatively expensive with limited availability in resource-poor settings.

VTE events occurred antepartum. There were no events in the postpartum period despite the associated higher risk of VTE.^26,45^ In intermediate-risk pregnancies, postpartum thromboprophylaxis was extended beyond the recommended 2 weeks in the presence of ongoing risk factors, for a median of 4 (4) weeks. High-risk pregnancies received postpartum thromboprophylaxis, as recommended, for a median of 6 (0) weeks. In the peripartum period, pregnancies were predominantly managed with scheduled delivery. Similar studies, however, have reported an increased risk of postpartum VTE and have as such proposed expectant management of labor to minimize the interruption of anticoagulation.^46,47^ Nevertheless, concerns exist over the risks of spontaneous labor with the use of intermediate prophylactic or therapeutic enoxaparin.^27^ Further prospective studies are indicated to determine the safety of expectant management of labor in women receiving higher doses of LMWH.

In this study, recurrent VTE occurred in women with the following combinations of risk factors: SLE, non-criteria antiphospholipid syndrome and previous pregnancy-related PE; HIV and previously unprovoked DVT; and multiple low to intermediate risk factors. With regards to non-criteria antiphospholipid syndrome, the presence of antiphospholipid antibodies on the same occasion has been associated with recurrent thrombosis. According to a recent prospective study, the presence of 2 or more types of antiphospholipid antibodies on the same/different occasions were also predictors of recurrence in participants with a previously unprovoked VTE (HR: 4.5, 95% CI: 1.5-13.0).^48^ Furthermore, in pregnant women living with HIV, we have previously shown at least a 2-fold increased risk of VTE, independent of HIV viral load, CD4 count, and ART.^19^ Current guidelines, however, do not consider HIV as an indication for antepartum or postpartum thromboprophylaxis.^20,21,32,49^ Accordingly, the most recent American Society of Haematology guideline advises against thromboprophylaxis in women with a single clinical risk factor owing to the low level of evidence.^21^ Nonetheless, owing to the small number of VTE events, this study could not assess predictors of recurrent thrombosis in order to guide VTE prevention.

Bleeding is an important safety concern in pregnant women receiving thromboprophylaxis. The incidence of bleeding in this cohort was 7.8%, of which major bleeding events accounted for 3.1%. This is consistent with similar studies. Most recently, major bleeding rates of 4.0% were reported in pregnant women with prior VTE randomized to intermediate dose and fixed low dose LMWH respectively by investigators of the multi-center, multi-national Highlow study.^24^ Severe PPH rates ranging from 2.0% to 14.1% have been reported previously.^26–28,42,50^ Caution, however, is required when interpreting these studies, because of the use of nonstandardized definitions of bleeding.

This study evaluated predictors of bleeding, namely high-risk group, concomitant aspirin, comorbid HIV, pre-eclampsia and FGR, cesarean delivery, spontaneous delivery, and peripartum enoxaparin interval. On univariate analysis, no independent predictors of bleeding were identified. Of note, cesarean delivery was not a risk factor for bleeding, despite the rate of cesarean deliveries of 64.7% at this specialist referral center for high-risk pregnancies. Furthermore, management with anti-Xa-adjusted enoxaparin in high-risk pregnancies was also not an independent predictor of bleeding. Future prospective studies are indicated to identify predictors of bleeding using standardized bleeding definitions.

Several limitations of this study warrant consideration. Firstly, the high-risk group was heterogeneous with regards to enoxaparin dosing and also included pregnancies with extended duration anticoagulation (n = 9) who were prescribed therapeutic dose enoxaparin. These pregnancies were included as there is limited data on the associated risks of recurrent thrombosis and bleeding in African women. Secondly, bleeding was determined by visual estimation, which is increasingly recognized as an unreliable measure of bleeding. This may have resulted in an underestimation of bleeding events. Lastly, this study could not assess predictors of VTE as a result of the small number of recurrent events. In particular, HIV is a recognized risk factor for thrombosis, which merits further investigation.

In summary, this study adds to the current literature about the risks related to thrombosis and bleeding associated with antepartum and postpartum thromboprophylaxis in intermediate- and high-risk pregnancies. In this cohort, managed predominantly with scheduled delivery, there were no postpartum thrombosis events or reduced access to neuro-axial anesthesia. The rates of thrombosis and bleeding in this population of predominantly African ethnicity were consistent with similar studies and can be used to inform pregnant women of the benefits of anticoagulation and the risks of potential bleeding.
